# Comprehensive Learning Fungal Growth Optimizer for Numerical Optimization and Reservoir Production Optimization

**DOI:** 10.3390/biomimetics11060370

**Published:** 2026-05-27

**Authors:** Mingyang Gong, Zhenyu Song, Xiaonan Zhang, Yi Tang

**Affiliations:** 1 School of Geophysics and Petroleum Resources, Yangtze University, Wuhan 430100, China; 2021730024@yangtzeu.edu.cn (M.G.); 2021710289@yangtzeu.edu.cn (X.Z.); tangyi.stu@yangtzeu.edu.cn (Y.T.); 2 School of Earth Sciences, Yangtze University, Wuhan 430100, China

**Keywords:** Fungal Growth Optimizer, Comprehensive Learning Strategy, production optimization, global optimization

## Abstract

The Fungal Growth Optimizer (FGO) is a nature-inspired metaheuristic that simulates fungal colony behaviors, but its exploitation phase can lose search diversity when guidance is dominated by limited peer or global-best information. In this paper, we propose an enhanced variant called the Comprehensive Learning Fungal Growth Optimizer (CLFGO). We integrate a conditionally activated Comprehensive Learning (CL) strategy into the FGO framework. When a candidate solution stagnates, the strategy constructs a dimension-specific learning exemplar. This mechanism allows each dimension to learn from the personal best of a different peer, extending the original fungal growth model. CLFGO is therefore intended for high-dimensional, multimodal, hybrid, and composition landscapes in which the original FGO is prone to diversity loss, rather than as a universal replacement for all problem classes. This approach improves population diversity and reduces the risk of premature convergence. We evaluated CLFGO on 29 CEC2017 benchmark functions at 30 dimensions against nine metaheuristics under the same maximum-function-evaluation budget. CLFGO achieved the lowest mean error on 21 of 29 functions and attained a Friedman average rank of 1.5517. Furthermore, we applied CLFGO to a reservoir production optimization problem, where it obtained a mean Net Present Value of $9.97\times 10^{8}$ USD, outperforming the compared algorithms in both solution accuracy and convergence stability.

## 1. Introduction

Optimization problems frequently arise in engineering design, economic planning, and scientific research. Classical optimization techniques, such as gradient descent and the simplex method, have well-established theoretical foundations but can struggle with high-dimensional, nonlinear, or discontinuous objective functions [1,2,3,4,5,6].

Metaheuristic algorithms are increasingly used for these complex optimization tasks [7,8]. As stochastic, population-based methods, they require no assumptions about differentiability and can search diverse regions of the solution landscape to balance exploration and exploitation [9,10,11]. Recent developments also include surrogate-assisted metaheuristics for computationally expensive applications [12,13].

Modern metaheuristics are typically categorized into Evolutionary Algorithms (EAs) and Swarm Intelligence (SI) algorithms [14]. EAs, such as the Genetic Algorithm (GA) [15] and Differential Evolution (DE) [16], are based on principles of selection, crossover, and mutation. SI algorithms, such as Particle Swarm Optimization (PSO) [17,18] and Ant Colony Optimization (ACO) [19], model the collective intelligence of decentralized biological systems [20,21]. Contemporary strategies frequently hybridize these mechanisms to address complex optimization tasks [22,23].

The No Free Lunch (NFL) theorem for optimization [24] states that no single algorithm performs best on all possible problems. An algorithm that excels on one class of functions may underperform on another [25]. This principle motivates the ongoing development of specialized metaheuristics designed for specific problem characteristics, as aligning algorithmic mechanics with the target problem structure can yield significant practical benefits [26,27,28].

This study focuses on enhancing the Fungal Growth Optimizer (FGO), a bio-inspired algorithm that models candidate solutions as a network of hyphae and simulates fungal colony behaviors such as tip growth, chemotropism, branching, and spore germination. This multi-phase search process provides broad exploratory coverage [29,30]. However, the guidance mechanism in FGO has a structural limitation: during exploitation, individuals move primarily toward the global best solution or randomly selected peers. Because learning sources are not adapted dynamically, the algorithm lacks a mechanism for systematic knowledge sharing across the population [31]. Consequently, FGO can rapidly lose diversity and stagnate at local optima in complex multimodal spaces [32,33].

This structural diagnosis also clarifies the problem classes for which CLFGO is theoretically expected to be useful. The proposed mechanism is most relevant to high-dimensional, multimodal, hybrid, and composition problems where useful search information may be distributed across different individuals and dimensions, and where a single dominant attractor can cause premature loss of population diversity. In contrast, we do not claim that CLFGO should be universally superior on simple unimodal, low-dimensional, or single-basin problems; the No Free Lunch theorem implies that such universal dominance is not expected.

To address this limitation, we propose the Comprehensive Learning Fungal Growth Optimizer (CLFGO). We integrate a dimension-wise Comprehensive Learning (CL) module into the baseline FGO framework to facilitate cooperative behavior. When an individual stagnates, the CL module constructs a tailored learning exemplar through tournament-based peer selection, enabling the individual to learn from multiple peers rather than a single global attractor. This conditional activation preserves the rapid exploitation capabilities of the original hyphal growth operators while introducing necessary diversity. We evaluated CLFGO on 29 CEC2017 benchmark functions against nine metaheuristics. Furthermore, we applied CLFGO to a high-dimensional reservoir production optimization problem under geological uncertainty.

The main contributions of this work are summarized as follows:

- We identify the single-source guidance tendency of FGO as a key structural reason for diversity loss and clarify that CLFGO targets problems with multimodal, hybrid, composition, or dimension-wise heterogeneous structures.
- We adapt the Comprehensive Learning strategy as a conditionally activated module in FGO, so stagnant individuals can build dimension-wise exemplars from multiple high-quality peers without replacing the original fungal growth operators.
- We revise the experimental protocol to use a strict maximum-function-evaluation budget and provide parameter settings, diagnostic convergence/diversity evidence, and time–space complexity analysis for fairer comparison.

The remainder of this paper is organized as follows. Section 2 describes the original FGO algorithm. Section 3 presents the proposed Comprehensive Learning strategy and the CLFGO framework. Section 4 details the experimental setup and benchmark comparisons. Section 5 applies CLFGO to a reservoir production optimization case study. Section 6 concludes the paper.

## 2. Original Algorithm

The Fungal Growth Optimizer (FGO), proposed by Abdel-Basset et al. [32], is a nature-inspired metaheuristic that simulates the growth behaviors of fungal colonies. It is built on three core biological mechanisms: hyphal tip growth (comprising exploration and exploitation phases), hyphal branching, and spore germination.

### 2.1. Mathematical Model of FGO

The algorithm initializes a population of *N* candidate solutions (hyphae) and updates their positions through the following mechanisms.

**1. Initial Population Generation:** At the start, *N* hyphae (solutions) are randomly distributed within the *D*-dimensional search space bounded by lower ($\vec{S_{L}}$) and upper ($\vec{S_{U}}$) limits. This simulates the random germination of spores in a suitable environment.
  (1) $$\vec{S_{i}}=\vec{S_{L}}+\vec{r}⊙\left(\vec{S_{U}}-\vec{S_{L}}\right),\;i=1,2,\ldots ,N,$$
  where $\vec{r}$ is a vector of random numbers uniformly distributed in [0,1] and ⊙ denotes the Hadamard product.
**2. Hyphal Tip Growth:** This mechanism models the elongation and directional change of a hypha. It is divided into an exploration phase and an exploitation phase, controlled by a normalized fitness probability $p_{i}$ and a decaying exploration rate $E_{r}$. The growth rate *E* and direction $\vec{D}$ are calculated using the fitness of solutions and random peer selection.
  (2) $$\vec{S_{i}^{t+1}}=\left\{\begin{array}{cc} \vec{S_{i}^{t}}+E\cdot \vec{D}, & \mathrm{if}\;p_{i}<E_{r}\;(\mathrm{Exploration}\;\mathrm{Phase}\;I)\\ \mathrm{perform}\;\mathrm{local}\;\mathrm{chemotropism}\;\mathrm{search}, & \mathrm{otherwise}\;(\mathrm{Exploitation}\;\mathrm{Phase}\;I) \end{array}\right.$$
  where $E=e^{F}$ with $F=\frac{f_{i}}{\sum_{k=1}^{N}f_{k}}\cdot r_{1}\cdot E$, $\vec{D}=\vec{S_{t_{a}}}-\vec{S_{t_{c}}}$, and $p_{i}$ is the normalized fitness of the *i*-th solution. E represents the environmental factor, $r_{1}$ is a uniformly distributed random number in [0,1], and $\vec{S_{t_{a}}}$, $\vec{S_{t_{c}}}$ are randomly chosen mutually exclusive peer individuals from the current population. The exploitation phase involves chemotropism, where a hypha moves toward either a randomly selected peer or the global best solution, with an additional exploratory step to avoid premature convergence.
**3. Hyphal Branching:** This behavior enhances exploration by generating a new solution (branch) from an existing one. The new position is influenced by the difference between two randomly chosen solutions and the difference between a random solution and the global best.
  (3) $$S_{i,j}^{t+1}=\left[R_{j}<r_{3}\right]\cdot S_{i,j}^{t}+\left(1-\left[R_{j}<r_{3}\right]\right)\cdot \left(S_{i,j}^{t}+r_{5}\cdot E_{L}\cdot D_{i,j}^{ep1}+\left(1-r_{5}\right)\cdot E_{L}\cdot D_{i,j}^{ep2}\right),$$
  where $D_{i,j}^{ep1}=S_{a,j}^{t}-S_{b,j}^{t}$, $D_{i,j}^{ep2}=S_{c,j}^{t}-S_{j}^{*}$, $E_{L}=1+e^{\frac{f_{i}}{\sum_{k=1}^{N}f_{k}}}\cdot \left[r_{6}<r_{7}\right]$, and [·] is the Iverson bracket. Here, $R_{j},r_{3},r_{5},r_{6},$ and $r_{7}$ denote randomly generated numbers in [0,1], $S_{j}^{*}$ represents the *j*-th dimension of the global best position, and $S_{a,j}^{t},S_{b,j}^{t},S_{c,j}^{t}$ are elements of three distinct randomly selected individuals.
**4. Spore Germination:** This mechanism re-initializes solutions in new areas to escape local optima. Initially, spores land in random positions (pure exploration). As the search progresses, they land between the global best and a random position, promoting exploitation.
  (4) $$\begin{array}{cc} S_{i,j}^{t+1} & =\left[R_{j}<r_{3}\right]\cdot S_{i,j}^{t}+\left(1-\left[R_{j}<r_{3}\right]\right)\cdot (\frac{\left(\left(\frac{t}{t_{\max}}\right)\cdot S_{j}^{*}+\left(1-\frac{t}{t_{\max}}\right)\cdot S_{a,j}^{t}\right)}{2}+S_{b,j}^{t}\\ \; & \;+S_{g}\cdot r_{5}\cdot E\cdot \left|\frac{S_{c,j}^{t}+S_{a,j}^{t}+S_{b,j}^{t}}{3}-S_{i,j}^{t}\right|), \end{array}$$
  where $S_{g}$ is randomly chosen as −1 or 1, *t* is the current iteration, and $t_{\max}$ is the maximum number of iterations used to compute the normalized search progress.

### 2.2. Integrated Iteration

The complete FGO algorithm initializes a population of *N* hyphae using Equation (1). In each iteration, the algorithm first stochastically selects between executing the *Hyphal Tip Growth* loop and the *Branching-Germination* loop based on a random comparison. Within the selected loop, each individual is updated according to the corresponding mathematical model. After a new candidate position is generated and evaluated, the function-evaluation counter is increased by one. A greedy selection is then performed: the new position replaces the current one if and only if it yields improved fitness. This process repeats until the maximum number of function evaluations (MaxFEs) is reached, at which point the global best solution $\vec{S^{*}}$ is returned as the final output.

### 2.3. Pseudocode of the Original FGO

The pseudocode for the original FGO algorithm is presented in Algorithm 1, summarizing the initialization, main loop, and update procedures.

**Algorithm 1** Original Fungal Growth Optimizer (FGO)
  1:**Input:** Population size *N*, maximum function evaluations MaxFEs, problem dimension *D*, bounds $\vec{S_{L}},\vec{S_{U}}$  2:**Initialize** *N* hyphae $\vec{S_{i}}$ using Equation (1)  3:Evaluate fitness $f(\vec{S_{i}})$ and identify the global best $\vec{S^{*}}$  4:FEs←N, t←1  5:**while** FEs<MaxFEs **do**  6:    Generate random numbers $r_{9},r_{10}∼\mathrm{rand}\left(0,1\right)$  7:    **if** $r_{9}<r_{10}$ **then**                       ▹ Execute Hyphal Tip Growth loop  8:        **for** i=1 to *N* **do**  9:           **if** FEs≥MaxFEs **then**10:               **break**11:           **end if**12:           Compute exploration rate $E_{r}$ and normalized fitness probability $p_{i}$13:           **if** $p_{i}<E_{r}$ **then**14:               Update $\vec{S_{i}^{t+1}}$ via Exploration Phase I (Equation (2))15:           **else**16:               Update $\vec{S_{i}^{t+1}}$ via Exploitation Phase I (Equation (2))17:           **end if**18:           Evaluate $\vec{S_{i}^{t+1}}$ and set FEs←FEs+119:           Greedy selection: if $f\left(\vec{S_{i}^{t+1}}\right)<f\left(\vec{S_{i}^{t}}\right)$, set $\vec{S_{i}^{t}}\leftarrow \vec{S_{i}^{t+1}}$20:        **end for**21:    **else**                        ▹ Execute Branching and Germination loop22:        **for** i=1 to *N* **do**23:           **if** FEs≥MaxFEs **then**24:               **break**25:           **end if**26:           Generate random number $r_{7}∼\mathrm{rand}\left(0,1\right)$27:           **if** $r_{7}<0.5$ **then**28:               Update $\vec{S_{i}^{t+1}}$ via Hyphal Branching (Equation (3))29:           **else**30:               Update $\vec{S_{i}^{t+1}}$ via Spore Germination (Equation (4))31:           **end if**32:           Evaluate $\vec{S_{i}^{t+1}}$ and set FEs←FEs+133:           Greedy selection: if $f\left(\vec{S_{i}^{t+1}}\right)<f\left(\vec{S_{i}^{t}}\right)$, set $\vec{S_{i}^{t}}\leftarrow \vec{S_{i}^{t+1}}$34:        **end for**35:    **end if**36:    Update global best $\vec{S^{*}}$ if a better solution is found37:    t←t+138:
**end while**
39:**Output:** Global best solution $\vec{S^{*}}$


## 3. The Proposed CLFGO Algorithm

This section describes the core components of CLFGO and the integration of the Comprehensive Learning (CL) strategy into the FGO framework. The CL module provides stagnant individuals with multi-source, dimension-wise learning guidance to reduce the risk of premature convergence.

### 3.1. Comprehensive Learning Strategy (CLS)

The Comprehensive Learning Strategy (CLS) is inspired by dimension-wise multi-exemplar learning methods such as CL-PSO. We adapt this strategy as a conditionally activated mechanism within the FGO structure. It diversifies search directions by allowing individuals to learn from different peers for each dimension, reducing reliance on a single global attractor.

#### 3.1.1. Triggering Condition and Exemplar Vector

The strategy is conditionally activated when an individual *i* is identified as *stagnant*, meaning that its personal best solution has not improved over a predefined number of consecutive iterations. For a stagnant individual, a new *learning exemplar vector* is constructed as:

(5) $$e_{i}=\left[e_{i}\left(1\right),e_{i}\left(2\right),\ldots ,e_{i}\left(D\right)\right],$$

where *D* is the problem dimensionality. Each element $e_{i}\left(j\right)$ specifies the index of the exemplar individual from which particle *i* learns in the *j*-th dimension.

#### 3.1.2. Learning Probability

Each individual is assigned a learning probability $P_{c_{i}}$ that controls its propensity to learn from others versus itself. This probability is defined by a monotonically increasing function based on the individual’s index within the sorted population:

(6) $$P_{c_{i}}=a+b\times \frac{\exp\left(10\cdot \frac{i-1}{N}\right)-1}{\exp(10)-1}.$$

Here, *N* is the population size, *i* is the index of the current individual, and *a* and *b* are the lower and upper bounds for the learning probability, respectively. We set a=0 and b=0.5 as conservative CLPSO-derived bounds [34], not as a newly optimized universal setting. This choice restricts the maximum probability of external exemplar learning to 0.5, so each individual still retains substantial influence from its own historical best position while stagnant individuals can receive cross-dimensional guidance.

#### 3.1.3. Dimension-Wise Exemplar Selection

For each dimension j∈{1,2,…,D}, the exemplar index $e_{i}\left(j\right)$ is determined through a stochastic tournament process. A random number rand∈[0,1] is drawn. If $\mathrm{rand}<P_{c_{i}}$, the individual learns from external peers: two distinct individuals $k_{1}$ and $k_{2}$ (with $k_{1},k_{2}\neq i$) are randomly selected, and the one with the better historical best fitness is chosen as the exemplar for that dimension. Otherwise (i.e., $\mathrm{rand}\geq P_{c_{i}}$), the individual retains its own historical best position. This mechanism is formally expressed as:

(7) $$e_{i}\left(j\right)=\left\{\begin{array}{cc} \arg\min_{k\in \{k_{1},k_{2}\}}pfit\left(k\right), & \mathrm{if}\;\mathrm{rand}<P_{c_{i}},\\ i, & \mathrm{if}\;\mathrm{rand}\geq P_{c_{i}}. \end{array}\right.$$

#### 3.1.4. Safeguard Mechanism

To prevent degenerate learning where an individual learns exclusively from itself, a safeguard is applied. After constructing $e_{i}$, the algorithm checks if:

(8) $$e_{i}\left(j\right)=i,\;\forall j\in \left\{1,2,\ldots ,D\right\}.$$

If Equation (8) holds, a random dimension $j^{\prime }$ is selected, and a distinct individual $k_{3}\neq i$ is randomly chosen, such that $e_{i}\left(j^{\prime }\right)=k_{3}$. This ensures that every stagnant individual learns from at least one external peer.

### 3.2. Implementation Framework of CLFGO

**Figure 1:**
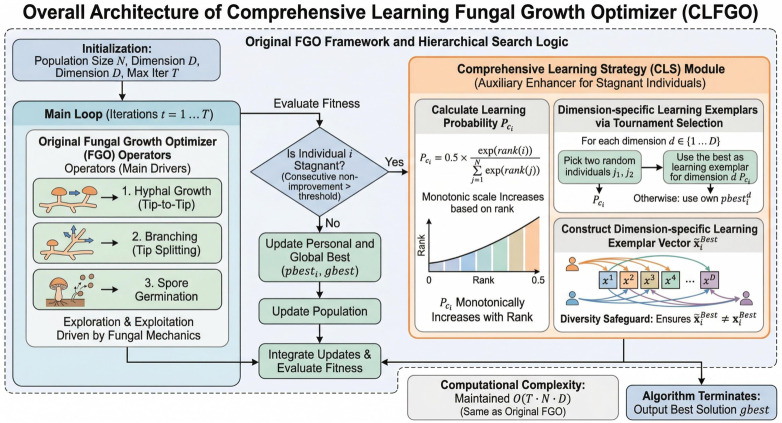
Flowchart of the proposed CLFGO algorithm.

CLFGO integrates the CLS as a conditionally activated module within the original FGO workflow, as illustrated in Figure 1. During the main iterative loop, the algorithm monitors for stagnant individuals. For stagnant individual *i*, the CLS module is invoked before the standard FGO update. It generates a new candidate position using the exemplar vector $e_{i}$. If this CL-generated position yields improved fitness, it is accepted. The individual then executes the standard FGO operators from this updated position. This mechanism introduces new search directions for trapped solutions without replacing the original bio-inspired dynamics.

### 3.3. Pseudocode of the Proposed CLFGO

Algorithm 2 presents the pseudocode for CLFGO. The procedure integrates the CLS as an inner loop executed before the standard FGO update operators. At each iteration, the algorithm checks whether each individual has stagnated (Lines 7–8). For a stagnant individual, the CLS module constructs an exemplar vector (Lines 9–18) according to the learning probability $P_{c_{i}}$ (Equation (6)) and the tournament selection rule (Equation (7)). The safeguard mechanism (Lines 19–21) ensures that at least one dimension learns from an external peer. A new candidate position is generated from the exemplar vector and evaluated via greedy selection (Lines 22–24). The individual then executes the standard FGO operators. Each objective evaluation produced by either the CL module or the standard FGO operators is counted toward the same MaxFEs budget. This structure injects diversity into trapped solutions while preserving the original search dynamics.
**Algorithm 2** Comprehensive Learning Fungal Growth Optimizer (CLFGO)  1:**Input:** Population size *N*, maximum function evaluations MaxFEs, dimension *D*, bounds $\vec{S_{L}},\vec{S_{U}}$, stagnation threshold *m*, learning bounds a,b  2:**Initialize** *N* hyphae $\vec{S_{i}}$ using Equation (1); evaluate fitness; identify $\vec{S^{*}}$  3:Initialize personal best positions $\vec{pbest_{i}}\leftarrow \vec{S_{i}}$, stagnation counters $sc_{i}\leftarrow 0$, ∀i  4:Compute learning probabilities $P_{c_{i}}$ using Equation (6), ∀i  5:FEs←N, t←1  6:**while** FEs<MaxFEs **do**  7:    **for** i=1 to *N* **do**                                               ▹ **CLS Module**  8:        **if** FEs≥MaxFEs **then**  9:           **break**10:        **end if**11:        **if** $sc_{i}\geq m$ **then**                                           ▹ Individual *i* is stagnant12:           **for** j=1 to *D* **do**                                         ▹ Build exemplar vector $e_{i}$13:               Generate rand∼rand(0,1)14:               **if** $\mathrm{rand}<P_{c_{i}}$ **then**15:                   Randomly select $k_{1},k_{2}\neq i$16:                   $e_{i}\left(j\right)\leftarrow \arg\min_{k\in \{k_{1},k_{2}\}}pfit\left(k\right)$                                  ▹ Tournament selection17:               **else**18:                   $e_{i}\left(j\right)\leftarrow i$                                           ▹ Learn from own best19:               **end if**20:           **end for**21:           **if** $e_{i}\left(j\right)=i,\;\forall j$ **then**                                            ▹ Safeguard check22:               Randomly select dimension $j^{\prime }$ and individual $k_{3}\neq i$23:               $e_{i}\left(j^{\prime }\right)\leftarrow k_{3}$24:           **end if**25:           Construct candidate $\vec{S_{i}^{\mathrm{CL}}}$: for each dimension *j*, set $S_{i,j}^{\mathrm{CL}}\leftarrow pbest_{e_{i}\left(j\right),j}$26:           Evaluate $\vec{S_{i}^{\mathrm{CL}}}$ and set FEs←FEs+127:           **if** $f\left(\vec{S_{i}^{\mathrm{CL}}}\right)<f\left(\vec{S_{i}^{t}}\right)$ **then** $\vec{S_{i}^{t}}\leftarrow \vec{S_{i}^{\mathrm{CL}}}$; $sc_{i}\leftarrow 0$28:        **end if**29:    **end for**30:                                          ▹ **Standard FGO Update (Algorithm 1, Lines 6–16)**31:    Execute Hyphal Tip Growth **or** Branching-Germination loop on all individuals while respecting MaxFEs32:    Perform greedy selection for each individual; update $\vec{pbest_{i}}$ and $sc_{i}$ accordingly33:    Update global best $\vec{S^{*}}$ if a better solution is found34:    t←t+135:**end while**36:**Output:** Global best solution $\vec{S^{*}}$

### 3.4. Complexity Analysis

For the original FGO, the arithmetic update cost per iteration is O(N·D), and the cumulative update cost over *T* iterations is O(T·N·D), excluding the objective-function evaluation cost. In CLFGO, let $q_{t}$ denote the number of stagnant individuals at iteration *t*. The CL module constructs dimension-wise exemplar vectors only for these stagnant individuals, yielding an additional $O(q_{t}\cdot D)$ arithmetic cost and at most $q_{t}$ additional objective evaluations. Since $q_{t}\leq N$, the worst-case auxiliary update cost remains O(N·D) per iteration, and all additional objective evaluations are counted under the same MaxFEs stopping budget used by the compared algorithms.

The space complexity also remains linear in N·D, but CLFGO requires additional storage beyond the original FGO. Specifically, FGO stores the current population and related fitness values, whereas CLFGO additionally stores personal-best positions, exemplar indices, stagnation counters, and learning probabilities. Table 1 summarizes the resulting time and memory requirements.

To quantify the runtime overhead of the proposed CL module, we profiled CLFGO, FGO, and the nine compared algorithms on the unimodal function F1. A full 29-function, 30-run runtime profile would be expensive, so the profiling was run over 10 independent trials under the same settings (population size N=30, dimension D=30, and MaxFEs=300,000) in MATLAB R2022b on an Intel Core i7-10700 CPU at 2.90 GHz. The total execution times are summarized in Table 2.

The results in Table 2 show that CLFGO adds a modest runtime overhead (approximately 18%) compared with FGO. This cost comes from stagnation checking, personal-best tracking, and dimension-wise exemplar construction, which is activated only for stagnant individuals. In this setting, CLFGO is close to MGO and faster than HGS, SMA, and PO. These timings are consistent with the stated O(T·N·D) update cost and indicate that the CL module adds limited overhead relative to the full run.

## 4. Experimental Results and Analysis

This section reports the empirical evaluation of CLFGO against nine metaheuristics on CEC2017 benchmark functions and a reservoir production optimization problem.

### 4.1. Experimental Setup

All algorithms were implemented and evaluated under the same MATLAB execution environment. The population size was N=30, and the problem dimensionality was D=30. The search terminated at the same maximum-function-evaluation budget for every algorithm, namely MaxFEs=10,000×D=300,000 for the 30-dimensional CEC2017 experiments. Any objective evaluation generated by the CL module was counted toward this budget; therefore, CLFGO was not allowed extra evaluations beyond the competing algorithms. We performed 30 independent runs for each function using uniformly distributed random seeds and recorded the mean and standard deviation of the best fitness values.

The internal parameters for all compared algorithms were set to the default values recommended in their original publications (Table 3). For CLFGO, the learning probability bounds were set to a=0 and b=0.5 (Equation (6)). The upper bound of 0.5 allows individuals to retain information from their own historical best positions. The stagnation threshold was set to m=5.

### 4.2. Sensitivity Analysis of the Stagnation Threshold

The stagnation threshold *m* controls when the CL strategy is activated. A small value triggers external learning too frequently and may disturb useful FGO exploitation, whereas a large value delays the rescue of stagnant individuals. To test this parameter more broadly, five CLFGO variants with stagnation thresholds m∈{1,3,5,7,10} were compared on the entire CEC2017 benchmark suite. The statistical results obtained across 30 dimensions are summarized in Table 4.

The results in Table 4 show that CLFGO with m=5 gives the best overall outcome among the tested settings. When the stagnation threshold is very small (m=1), comprehensive learning is triggered too often and disrupts the baseline FGO search, giving a mean rank of 4.10 and 19 losses. Larger thresholds (m=7 and m=10) remain close to m=5, with Wilcoxon tests showing no statistically significant differences (≈) on most benchmark functions. We therefore use m=5 as the default because it gives the highest best count and the lowest average rank among the tested values. This sensitivity analysis covers unimodal, multimodal, hybrid, and composition functions, and supports using m=5 across the tested landscape types. The learning-probability bounds a=0 and b=0.5 are retained as conservative CLPSO-derived values so that individuals keep substantial self-influence while receiving cross-dimensional guidance under stagnation.

### 4.3. Benchmark Functions Overview

CLFGO is tested on the CEC2017 benchmark suite for real-parameter numerical optimization [35,36]. Following the common CEC2017 practice, F2 is excluded, leaving 29 scalable, bound-constrained test functions. These include two unimodal functions (F1 and F3), seven multimodal functions (F4–F10), ten hybrid functions (F11–F20), and ten composition functions (F21–F30). The hybrid and composition groups feature complex landscapes with shifted and asymmetric optima. Together, these functions allow an assessment of an algorithm’s search characteristics. Function definitions and properties are listed in Table 5.

### 4.4. Performance Comparison with Other Algorithms

The proposed CLFGO is compared against nine established metaheuristics: Bat Algorithm (BA) [37], Moss Growth Optimization (MGO) [38], Parrot Optimizer (PO) [39], Fungal Growth Optimizer (FGO) [32], Differential Evolution (DE) [16], Hunger Games Search (HGS) [40], Grey Wolf Optimizer (GWO) [41], Slime Mould Algorithm (SMA) [42], and Sine Cosine Algorithm (SCA) [43]. All algorithms are executed under identical conditions, including the same population size, maximum number of function evaluations, and 30 independent runs per function. Before the full benchmark comparison, Table 6 provides a diagnostic comparison between CLFGO and the original FGO on representative CEC2017 functions. The mean (Avg) and standard deviation (Std) of the best-found fitness values are reported in Table 7.

CLFGO obtained the lowest mean error on 21 of 29 functions and achieved a Friedman average rank of 1.5517, followed by DE (3.3103) and MGO (3.3448). On hybrid and composition functions (e.g., F12, F13, and F21), CLFGO yielded mean errors lower than FGO. On simpler functions (e.g., F1 and F6), CLFGO, MGO, and DE achieved comparable final fitness values. Based on the pairwise +/=/− summary in Table 7, CLFGO performed better than HGS, SMA, and SCA on all 29 functions. Against MGO, CLFGO recorded 17 wins, 11 ties, and 1 loss. Against DE, CLFGO recorded 22 wins, 4 ties, and 3 losses (on F16, F17, and F20).

For the unimodal functions (F1 and F3), CLFGO converged to the global optimum on F1 with a standard deviation of $3.12\times 10^{-14}$. For multimodal functions (F4–F10), CLFGO and DE reached the theoretical optimum on F6 ($Avg=6.00\times 10^{2}$), and CLFGO yielded the lowest mean on F4, F7, and F10. DE achieved the lowest mean on F9. For hybrid functions (F11–F20), CLFGO ranked first on 7 of 10 instances. For example, on F12 and F13, CLFGO achieved $Avg=5.18\times 10^{3}$ and $1.35\times 10^{3}$, respectively. For composition functions (F21–F30), CLFGO obtained the lowest mean on several instances, while FGO and MGO achieved the lowest means on F22 and F24, respectively.

We applied the Wilcoxon signed-rank test (α=0.05) to assess statistical significance; Table 8 reports the *p*-values. CLFGO achieved p<0.05 against BA, PO, FGO, HGS, SMA, and SCA on most functions. Against MGO, non-significant *p*-values (p>0.05) occurred on 11 functions, including F5 and F6. Against DE, non-significant *p*-values occurred on 4 functions, including F16, F17, and F20.

This diagnostic comparison provides direct evidence for the motivation behind CLFGO on representative functions from different landscape classes. The large performance gaps on F1, F13, and F30 indicate that the original FGO can converge to inferior regions under the same evaluation budget, whereas the negative gap on F22 shows that CLFGO is not uniformly superior. This pattern is consistent with the No Free Lunch theorem and motivates a balanced interpretation of the results.

The practical effect size is therefore function-dependent. On difficult hybrid and composition cases such as F13 and F30, CLFGO provides large reductions in mean objective value relative to FGO. On F22, however, the original FGO obtains a lower mean value, suggesting that a single-attractor update can still be advantageous when the basin structure is compatible with the original fungal growth dynamics. Similarly, the non-significant comparisons with MGO and DE on several functions show that some practical differences are small even when CLFGO has the best overall rank.

**Figure 2:**
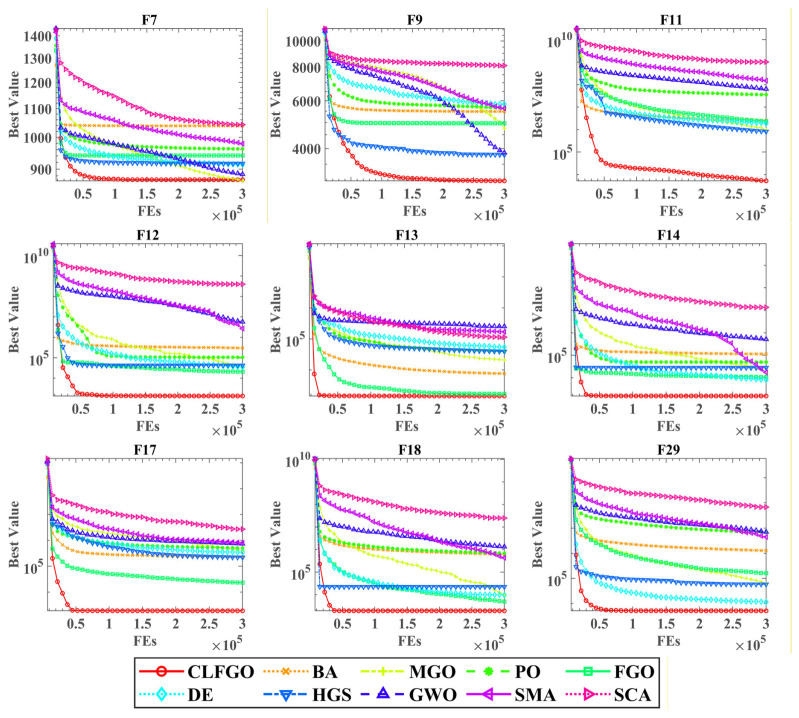
Convergence behavior analysis on nine representative benchmark functions. Each subplot corresponds to a specific test function, illustrating the decay of the objective value over the course of evaluations.

**Figure 3:**
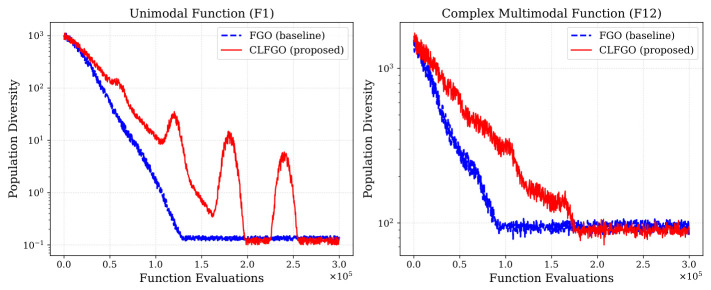
Population diversity trends of FGO and CLFGO across evaluations. The subplots illustrate the diversity metric over evaluations for representative functions (F1 and F12).

Figure 2 shows the convergence trajectories. CLFGO maintained continuous improvement in later stages without early stagnation. To evaluate population diversity across evaluations, we used the centroid-distance metric:

(9) $$Div=\frac{1}{N}\sum_{i=1}^{N}\sqrt{\sum_{j=1}^{D}{\left(x_{i,j}-{\bar{x}}_{j}\right)}^{2}}$$

where $x_{i,j}$ is the *j*-th dimension of the *i*-th individual, and ${\bar{x}}_{j}$ is the mean across the population for dimension *j*. Figure 3 illustrates the diversity profiles of FGO and CLFGO. The CLFGO trajectory exhibits diversity rebounds. When the population stagnates, the CL mechanism provides new directional guidance, whereas FGO loses diversity and converges prematurely.

## 5. Application to Production Optimization

Reservoir production optimization involves determining an operational strategy to maximize Net Present Value (NPV) over the life of a project. The problem is high-dimensional because optimal control variables, such as well rates or bottom-hole pressures, must be specified for multiple wells across discrete time intervals. Because reservoir simulation is nonlinear and computationally expensive, metaheuristic algorithms are an attractive alternative to classical gradient-based methods.

We deployed CLFGO within a simulation-based optimization framework. A synthetic multi-channel reservoir model served as the test case. We used the Eclipse simulator as the forward model to assess the physical and economic outcomes of each candidate control strategy. The algorithm optimizes well controls to manage waterflooding dynamics, with the objective of increasing hydrocarbon recovery while limiting water production and injection costs.

The economic performance of a given control strategy is quantified by the NPV objective function, defined as:

(10) $$NPV\left(x,z\right)=\sum_{t=1}^{n}\frac{\Delta t\cdot (Q_{o,t}r_{o}-Q_{w,t}r_{w}-Q_{i,t}r_{i})}{{\left(1+b\right)}^{p_{t}}}$$

where x and z represent the control variables and the reservoir state, respectively; $Q_{o,t}$, $Q_{w,t}$, and $Q_{i,t}$ denote the cumulative oil production, water production, and water injection rates at time step *t*; $r_{o}$, $r_{w}$, and $r_{i}$ are the associated unit prices (or costs) for oil, produced water, and injected water, respectively; *b* is the annual discount rate; and $p_{t}$ is the elapsed time in years. In this formulation, we treat the NPV optimization as an unconstrained problem to benchmark the numerical search performance of the algorithms.

### 5.1. Reservoir Model Description

**Figure 4:**
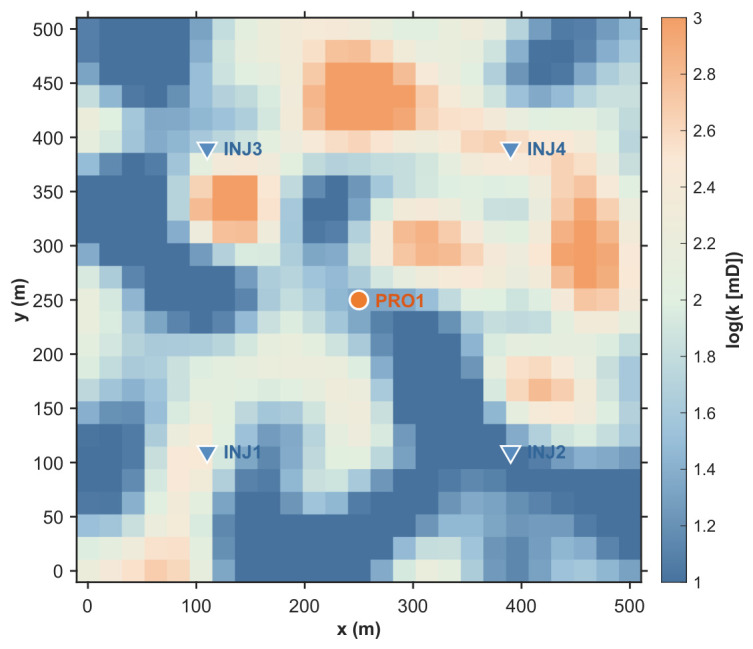
Two-dimensional synthetic reservoir model and well pattern arrangement.

We constructed a two-dimensional, heterogeneous synthetic reservoir model to represent a fluvial depositional environment. The model uses a five-spot well pattern with a single central production well (PRO1) and four corner injection wells (INJ1 through INJ4), as shown in Figure 4.

The domain was discretized on a 25×25 Cartesian grid. Each cell has uniform thickness and dimensions of 20 m by 20 m. Porosity was set to a constant value of 0.2. The permeability field was generated stochastically to produce a heterogeneous distribution with interconnected high-permeability channels embedded in a low-permeability matrix. Figure 4 shows the resulting log-permeability, ln(K), field.

The optimization framework was applied over a production horizon of 1800 days, partitioned into 6 discrete control steps. Optimization variables were assigned to all five wells, resulting in a problem dimension of 30 variables. Key economic parameters were set as follows: oil price (100 USD/STB), water injection cost (5 USD/STB), water production cost (5 USD/STB), and an annual discount rate (3%).

### 5.2. Analysis and Discussion of Experimental Results

We compared CLFGO with the original FGO and eight other metaheuristics (DE, MGO, HGS, PO, BA, GWO, SMA, and SCA). We ran each algorithm independently under identical conditions and recorded the mean NPV (Mean), standard deviation (Std), best, and worst outcomes.

As shown in Table 9, CLFGO achieved a mean NPV of $9.9742\times 10^{8}$ USD with a standard deviation of $1.353\times 10^{7}$. DE and MGO reached mean values of $9.536\times 10^{8}$ and $9.487\times 10^{8}$ USD, respectively. The remaining algorithms produced lower mean values with larger standard deviations.

**Figure 5:**
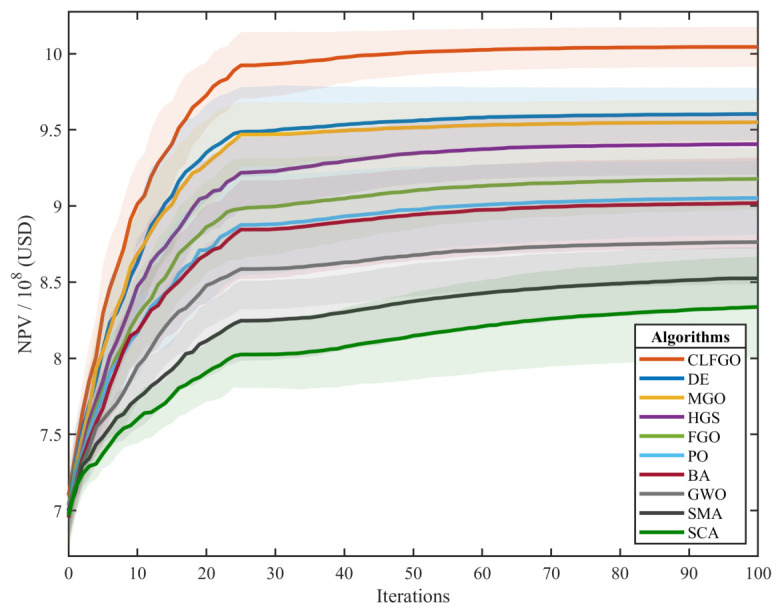
Convergence curve analysis of all algorithms on the production optimization problem.

The convergence trajectories in Figure 5 show the optimization progress. CLFGO reached its final NPV plateau earlier than the other algorithms, whereas DE and MGO converged at lower values.

Overall, CLFGO obtained higher NPV values with lower variance compared to the other tested metaheuristics on this production optimization problem.

## 6. Conclusions

This study proposed the Comprehensive Learning Fungal Growth Optimizer (CLFGO), an enhanced variant of FGO that incorporates a conditionally activated Comprehensive Learning (CL) strategy. When a candidate solution stagnates, the CL module constructs dimension-specific learning exemplars from the personal best positions of multiple peers. This approach provides new directional guidance and reduces the risk of premature convergence.

We evaluated CLFGO on 29 CEC2017 benchmark functions at 30 dimensions against nine metaheuristics. CLFGO achieved the lowest mean error on 21 functions and attained a Friedman average rank of 1.5517. We also applied CLFGO to a 30-variable reservoir production optimization problem, where it obtained a mean Net Present Value of $9.97\times 10^{8}$ USD. These results show that CLFGO is most effective on several multimodal, hybrid, and composition landscapes, but they do not imply universal superiority. The cases where FGO, MGO, or DE perform comparably or better indicate that the benefit of comprehensive learning depends on the landscape structure and parameter setting.

Future research could evaluate CLFGO on higher-dimensional problems and apply it to a broader set of practical engineering tasks, including multi-objective optimization. Further work should also include broader joint sensitivity analysis of the stagnation threshold and learning-probability bounds, as well as additional constrained reservoir-control cases.

## Figures and Tables

**Table 1:** Time and space complexity comparison of FGO and CLFGO.

Algorithm	Main Additional Operations	Space Requirement
FGO	Population update and greedy selection under the evaluation budget; update arithmetic is O(N·D) per iteration.	Population matrix, candidate solutions, fitness values, and global best; O(N·D).
CLFGO	FGO operations plus exemplar construction for $q_{t}$ stagnant individuals; auxiliary arithmetic is $O(q_{t}\cdot D)$ and worst-case O(N·D) per iteration.	FGO storage plus personal bests (N·D), exemplar indices (N·D), stagnation counters (*N*), and learning probabilities (*N*); still O(N·D) with a larger constant factor.

**Table 2:** Execution time (seconds) of competing algorithms for F1 over 10 independent runs.

Algorithm	Runtime over 10 Runs (s)
**CLFGO (Ours)**	**23.4**
FGO	19.8
DE	16.5
MGO	22.1
HGS	29.8
PO	37.4
BA	26.2
GWO	17.1
SMA	33.5
SCA	18.9

**Table 3:** Parameter settings for the compared algorithms.

Algorithm	Parameter Settings
CLFGO	a=0,b=0.5,m=5
FGO	$M=0.6,E_{p}=0.7,R=0.9$
DE	F=0.5,CR=0.2
MGO	$w=2,d_{1}=0.2,rec\_num=10,divide\_num=\dim/4$
HGS	l=0.03
PO	γ=1.5,c=0.2,F:S:C:O=1:1:1:1
BA	$f_{\min}=0,f_{\max}=2,\alpha =0.9,\gamma =0.9$
GWO	a=[2,0] (decreases linearly)
SMA	a=[2,0],vb=[−2,2],E=[0,2]
SCA	a=2 (decreases linearly)

**Table 4:** Sensitivity analysis results of the stagnation threshold *m* on CEC2017 (30D). “Best” signifies the number of functions where the variant outperformed others. “Mean Rank” denotes the average Friedman ranking. The “+/≈/–” column summarizes the Wilcoxon rank-sum test results (α=0.05) compared to the proposed CLFGO (m=5). The most favorable results are highlighted in bold.

Algorithm	Best	Mean Rank	+/≈/–
**CLFGO (m=5) (Proposed)**	**15**	**1.85**	—
CLFGO (m=7)	5	2.15	1/23/5
CLFGO (m=10)	4	2.25	1/22/6
CLFGO (m=3)	3	2.45	0/20/9
CLFGO (m=1)	2	4.10	0/10/19

**Table 5:** CEC2017 benchmark functions.

Function	Function Name	Class	Optimum
F1	Shifted and Rotated Bent Cigar Function	Unimodal	100
F3	Shifted and Rotated Zakharov Function	Unimodal	300
F4	Shifted and Rotated Rosenbrock’s Function	Multimodal	400
F5	Shifted and Rotated Rastrigin’s Function	Multimodal	500
F6	Shifted and Rotated Expanded Scaffer’s F6 Function	Multimodal	600
F7	Shifted and Rotated Lunacek Bi-Rastrigin Function	Multimodal	700
F8	Shifted and Rotated Non-Continuous Rastrigin’s Function	Multimodal	800
F9	Shifted and Rotated Lévy Function	Multimodal	900
F10	Shifted and Rotated Schwefel’s Function	Multimodal	1000
F11	Hybrid Function 1 (N = 3)	Hybrid	1100
F12	Hybrid Function 2 (N = 3)	Hybrid	1200
F13	Hybrid Function 3 (N = 3)	Hybrid	1300
F14	Hybrid Function 4 (N = 4)	Hybrid	1400
F15	Hybrid Function 5 (N = 4)	Hybrid	1500
F16	Hybrid Function 6 (N = 4)	Hybrid	1600
F17	Hybrid Function 6 (N = 5)	Hybrid	1700
F18	Hybrid Function 6 (N = 5)	Hybrid	1800
F19	Hybrid Function 6 (N = 5)	Hybrid	1900
F20	Hybrid Function 6 (N = 6)	Hybrid	2000
F21	Composition Function 1 (N = 3)	Composition	2100
F22	Composition Function 2 (N = 3)	Composition	2200
F23	Composition Function 3 (N = 4)	Composition	2300
F24	Composition Function 4 (N = 4)	Composition	2400
F25	Composition Function 5 (N = 5)	Composition	2500
F26	Composition Function 6 (N = 5)	Composition	2600
F27	Composition Function 7 (N = 6)	Composition	2700
F28	Composition Function 8 (N = 6)	Composition	2800
F29	Composition Function 9 (N = 3)	Composition	2900
F30	Composition Function 10 (N = 3)	Composition	3000

**Table 6:** Diagnostic comparison between CLFGO and the original FGO on representative CEC2017 functions.

Function	Landscape Class	CLFGO Mean	FGO Mean	Relative Change
F1	Unimodal	$1.00\times 10^{2}$	$2.62\times 10^{3}$	96.18% lower
F13	Hybrid	$1.35\times 10^{3}$	$2.07\times 10^{4}$	93.49% lower
F22	Composition	$2.83\times 10^{3}$	$2.30\times 10^{3}$	23.20% higher
F30	Composition	$5.16\times 10^{3}$	$1.61\times 10^{5}$	96.79% lower

**Table 7:** Results of the CLFGO and Other Algorithms on CEC2017 Benchmark Functions.

	**F1**		**F3**		**F4**	
**Algo.**	**Avg**	**Std**	**Avg**	**Std**	**Avg**	**Std**
CLFGO	$1.0000\times 10^{2}$	$3.1224\times 10^{-14}$	$3.0063\times 10^{2}$	$1.2162\times 10^{0}$	$4.2069\times 10^{2}$	$2.8216\times 10^{1}$
FGO	$2.6185\times 10^{3}$	$3.2117\times 10^{3}$	$3.0001\times 10^{2}$	$6.0714\times 10^{-3}$	$5.0936\times 10^{2}$	$3.6315\times 10^{1}$
BA	$5.7871\times 10^{5}$	$2.8969\times 10^{5}$	$3.0013\times 10^{2}$	$1.2475\times 10^{-1}$	$4.7587\times 10^{2}$	$3.8566\times 10^{1}$
MGO	$8.0227\times 10^{4}$	$7.1266\times 10^{4}$	$4.7192\times 10^{4}$	$8.2184\times 10^{3}$	$4.9016\times 10^{2}$	$1.4137\times 10^{1}$
PO	$5.9712\times 10^{7}$	$6.3476\times 10^{7}$	$4.9114\times 10^{3}$	$3.6644\times 10^{3}$	$5.2519\times 10^{2}$	$3.7885\times 10^{1}$
DE	$1.2465\times 10^{3}$	$2.6438\times 10^{3}$	$1.8776\times 10^{4}$	$4.7163\times 10^{3}$	$4.8979\times 10^{2}$	$9.0528\times 10^{0}$
HGS	$7.0059\times 10^{3}$	$5.2460\times 10^{3}$	$1.0356\times 10^{3}$	$2.6317\times 10^{3}$	$4.7948\times 10^{2}$	$3.3440\times 10^{1}$
GWO	$3.1471\times 10^{9}$	$2.3755\times 10^{9}$	$3.3884\times 10^{4}$	$1.0158\times 10^{4}$	$5.9220\times 10^{2}$	$7.5289\times 10^{1}$
SMA	$2.7439\times 10^{9}$	$1.1593\times 10^{9}$	$3.6615\times 10^{4}$	$7.6129\times 10^{3}$	$6.3196\times 10^{2}$	$6.5237\times 10^{1}$
SCA	$1.2267\times 10^{10}$	$1.7253\times 10^{9}$	$3.5714\times 10^{4}$	$6.7693\times 10^{3}$	$1.4070\times 10^{3}$	$2.4774\times 10^{2}$
	**F5**		**F6**		**F7**	
**Algo.**	**Avg**	**Std**	**Avg**	**Std**	**Avg**	**Std**
CLFGO	$5.6551\times 10^{2}$	$1.5657\times 10^{1}$	$6.0000\times 10^{2}$	$8.7542\times 10^{-5}$	$7.9017\times 10^{2}$	$9.5515\times 10^{0}$
FGO	$6.8698\times 10^{2}$	$3.0135\times 10^{1}$	$6.5051\times 10^{2}$	$7.2809\times 10^{0}$	$9.9622\times 10^{2}$	$7.2567\times 10^{1}$
BA	$8.1173\times 10^{2}$	$5.4997\times 10^{1}$	$6.7303\times 10^{2}$	$8.4768\times 10^{0}$	$1.5826\times 10^{3}$	$2.2143\times 10^{2}$
MGO	$5.6439\times 10^{2}$	$8.6187\times 10^{0}$	$6.0000\times 10^{2}$	$2.8616\times 10^{-4}$	$8.0374\times 10^{2}$	$1.4414\times 10^{1}$
PO	$7.2900\times 10^{2}$	$3.2296\times 10^{1}$	$6.5597\times 10^{2}$	$9.6543\times 10^{0}$	$1.1439\times 10^{3}$	$5.7275\times 10^{1}$
DE	$6.0625\times 10^{2}$	$9.3108\times 10^{0}$	$6.0000\times 10^{2}$	$2.1111\times 10^{-14}$	$8.4370\times 10^{2}$	$9.9951\times 10^{0}$
HGS	$6.1673\times 10^{2}$	$2.4442\times 10^{1}$	$6.0236\times 10^{2}$	$1.8238\times 10^{0}$	$8.7604\times 10^{2}$	$3.6968\times 10^{1}$
GWO	$6.0702\times 10^{2}$	$3.2027\times 10^{1}$	$6.0804\times 10^{2}$	$2.9029\times 10^{0}$	$8.6088\times 10^{2}$	$3.5516\times 10^{1}$
SMA	$7.1159\times 10^{2}$	$2.8875\times 10^{1}$	$6.4204\times 10^{2}$	$6.8119\times 10^{0}$	$1.0796\times 10^{3}$	$5.5986\times 10^{1}$
SCA	$7.7770\times 10^{2}$	$1.4535\times 10^{1}$	$6.4945\times 10^{2}$	$4.4785\times 10^{0}$	$1.1242\times 10^{3}$	$3.9541\times 10^{1}$
	**F8**		**F9**		**F10**	
**Algo.**	**Avg**	**Std**	**Avg**	**Std**	**Avg**	**Std**
CLFGO	$8.6878\times 10^{2}$	$1.3353\times 10^{1}$	$9.6523\times 10^{2}$	$8.2741\times 10^{1}$	$3.0367\times 10^{3}$	$2.5171\times 10^{2}$
FGO	$9.4138\times 10^{2}$	$2.2556\times 10^{1}$	$3.7809\times 10^{3}$	$5.7515\times 10^{2}$	$4.9612\times 10^{3}$	$5.8230\times 10^{2}$
BA	$1.0386\times 10^{3}$	$5.8998\times 10^{1}$	$1.2391\times 10^{4}$	$4.2309\times 10^{3}$	$5.4809\times 10^{3}$	$6.6000\times 10^{2}$
MGO	$8.6549\times 10^{2}$	$1.3013\times 10^{1}$	$9.4407\times 10^{2}$	$4.0522\times 10^{1}$	$4.7499\times 10^{3}$	$4.6335\times 10^{2}$
PO	$9.6217\times 10^{2}$	$2.5998\times 10^{1}$	$4.8895\times 10^{3}$	$6.8892\times 10^{2}$	$5.6530\times 10^{3}$	$6.7458\times 10^{2}$
DE	$9.1376\times 10^{2}$	$7.2919\times 10^{0}$	$9.0000\times 10^{2}$	$1.0342\times 10^{-13}$	$5.8382\times 10^{3}$	$3.1386\times 10^{2}$
HGS	$9.1667\times 10^{2}$	$2.9234\times 10^{1}$	$3.4154\times 10^{3}$	$1.0052\times 10^{3}$	$3.7864\times 10^{3}$	$6.3334\times 10^{2}$
GWO	$8.8478\times 10^{2}$	$1.3488\times 10^{1}$	$1.7883\times 10^{3}$	$5.8545\times 10^{2}$	$3.8627\times 10^{3}$	$4.3283\times 10^{2}$
SMA	$9.7919\times 10^{2}$	$1.9267\times 10^{1}$	$5.8268\times 10^{3}$	$1.0532\times 10^{3}$	$5.6326\times 10^{3}$	$5.6824\times 10^{2}$
SCA	$1.0430\times 10^{3}$	$1.5779\times 10^{1}$	$5.4439\times 10^{3}$	$1.0109\times 10^{3}$	$8.1121\times 10^{3}$	$3.4609\times 10^{2}$
	**F11**		**F12**		**F13**	
**Algo.**	**Avg**	**Std**	**Avg**	**Std**	**Avg**	**Std**
CLFGO	$1.1396\times 10^{3}$	$2.9169\times 10^{1}$	$5.1839\times 10^{3}$	$4.8496\times 10^{3}$	$1.3471\times 10^{3}$	$1.5072\times 10^{1}$
FGO	$1.2502\times 10^{3}$	$5.3899\times 10^{1}$	$2.2899\times 10^{6}$	$1.3651\times 10^{6}$	$2.0704\times 10^{4}$	$9.9764\times 10^{3}$
BA	$1.3172\times 10^{3}$	$5.9886\times 10^{1}$	$2.5551\times 10^{6}$	$2.2489\times 10^{6}$	$3.0351\times 10^{5}$	$1.6486\times 10^{5}$
MGO	$1.1772\times 10^{3}$	$2.4467\times 10^{1}$	$9.6367\times 10^{5}$	$5.4647\times 10^{5}$	$3.1574\times 10^{4}$	$2.2113\times 10^{4}$
PO	$1.3059\times 10^{3}$	$5.5515\times 10^{1}$	$3.5675\times 10^{7}$	$5.2930\times 10^{7}$	$1.0568\times 10^{5}$	$6.3400\times 10^{4}$
DE	$1.1632\times 10^{3}$	$2.1787\times 10^{1}$	$1.8651\times 10^{6}$	$9.4241\times 10^{5}$	$3.5372\times 10^{4}$	$2.1249\times 10^{4}$
HGS	$1.2010\times 10^{3}$	$3.1945\times 10^{1}$	$8.0344\times 10^{5}$	$6.2646\times 10^{5}$	$4.2522\times 10^{4}$	$2.6288\times 10^{4}$
GWO	$1.6985\times 10^{3}$	$5.1984\times 10^{2}$	$6.4165\times 10^{7}$	$9.3655\times 10^{7}$	$5.7469\times 10^{6}$	$4.3713\times 10^{6}$
SMA	$1.5857\times 10^{3}$	$1.1707\times 10^{2}$	$1.4945\times 10^{8}$	$1.0666\times 10^{8}$	$2.6827\times 10^{6}$	$4.3713\times 10^{6}$
SCA	$2.1585\times 10^{3}$	$3.9949\times 10^{2}$	$1.0162\times 10^{9}$	$2.7512\times 10^{8}$	$3.9721\times 10^{8}$	$1.1359\times 10^{8}$
	**F14**		**F15**		**F16**	
**Algo.**	**Avg**	**Std**	**Avg**	**Std**	**Avg**	**Std**
CLFGO	$1.4353\times 10^{3}$	$1.4754\times 10^{1}$	$1.5211\times 10^{3}$	$1.8680\times 10^{1}$	$2.1785\times 10^{3}$	$1.9776\times 10^{2}$
FGO	$1.6765\times 10^{3}$	$1.6433\times 10^{2}$	$1.0511\times 10^{4}$	$4.1576\times 10^{3}$	$2.8919\times 10^{3}$	$2.8358\times 10^{2}$
BA	$7.5921\times 10^{3}$	$4.2427\times 10^{3}$	$1.1284\times 10^{5}$	$5.8977\times 10^{4}$	$3.4022\times 10^{3}$	$4.5829\times 10^{2}$
MGO	$2.0743\times 10^{4}$	$1.6799\times 10^{4}$	$1.7591\times 10^{4}$	$1.3086\times 10^{4}$	$2.2031\times 10^{3}$	$1.4777\times 10^{2}$
PO	$3.9725\times 10^{4}$	$3.0240\times 10^{4}$	$4.9128\times 10^{4}$	$3.5657\times 10^{4}$	$3.0939\times 10^{3}$	$3.0875\times 10^{2}$
DE	$5.4598\times 10^{4}$	$4.1089\times 10^{4}$	$8.0324\times 10^{3}$	$6.0760\times 10^{3}$	$2.0778\times 10^{3}$	$1.6211\times 10^{2}$
HGS	$3.9166\times 10^{4}$	$3.0219\times 10^{4}$	$2.7673\times 10^{4}$	$1.6455\times 10^{4}$	$2.7487\times 10^{3}$	$2.8922\times 10^{2}$
GWO	$2.5305\times 10^{5}$	$3.1890\times 10^{5}$	$5.1330\times 10^{5}$	$9.8548\times 10^{5}$	$2.3731\times 10^{3}$	$2.4511\times 10^{2}$
SMA	$1.6881\times 10^{5}$	$1.0876\times 10^{5}$	$1.7247\times 10^{5}$	$6.4281\times 10^{5}$	$2.8088\times 10^{3}$	$3.3201\times 10^{2}$
SCA	$1.1362\times 10^{5}$	$6.5603\times 10^{4}$	$1.3411\times 10^{7}$	$1.1272\times 10^{7}$	$3.6244\times 10^{3}$	$1.7430\times 10^{2}$
	**F17**		**F18**		**F19**	
**Algo.**	**Avg**	**Std**	**Avg**	**Std**	**Avg**	**Std**
CLFGO	$1.8480\times 10^{3}$	$1.0555\times 10^{2}$	$1.8726\times 10^{3}$	$1.9099\times 10^{2}$	$1.9147\times 10^{3}$	$5.3481\times 10^{0}$
FGO	$2.2678\times 10^{3}$	$1.8486\times 10^{2}$	$2.3034\times 10^{4}$	$2.2942\times 10^{4}$	$4.9358\times 10^{3}$	$4.4045\times 10^{3}$
BA	$2.7794\times 10^{3}$	$3.3758\times 10^{2}$	$2.2400\times 10^{5}$	$1.2361\times 10^{5}$	$6.0405\times 10^{5}$	$2.9488\times 10^{5}$
MGO	$1.8838\times 10^{3}$	$6.5789\times 10^{1}$	$4.1333\times 10^{5}$	$2.5438\times 10^{5}$	$9.2284\times 10^{3}$	$7.2945\times 10^{3}$
PO	$2.2929\times 10^{3}$	$1.7937\times 10^{2}$	$5.0234\times 10^{5}$	$3.5019\times 10^{5}$	$6.8670\times 10^{5}$	$4.4668\times 10^{5}$
DE	$1.8311\times 10^{3}$	$4.9974\times 10^{1}$	$3.4824\times 10^{5}$	$2.2942\times 10^{5}$	$9.6917\times 10^{3}$	$5.6799\times 10^{3}$
HGS	$2.1973\times 10^{3}$	$2.3921\times 10^{2}$	$1.1893\times 10^{5}$	$1.6991\times 10^{5}$	$2.2311\times 10^{4}$	$2.2521\times 10^{4}$
GWO	$1.9739\times 10^{3}$	$1.4562\times 10^{2}$	$7.3615\times 10^{5}$	$8.2559\times 10^{5}$	$1.3495\times 10^{6}$	$5.5985\times 10^{6}$
SMA	$2.2596\times 10^{3}$	$2.1955\times 10^{2}$	$8.0325\times 10^{5}$	$1.0480\times 10^{6}$	$4.3189\times 10^{5}$	$5.5269\times 10^{5}$
SCA	$2.4009\times 10^{3}$	$1.2734\times 10^{2}$	$2.7108\times 10^{6}$	$1.1316\times 10^{6}$	$2.4890\times 10^{7}$	$1.4984\times 10^{7}$
	**F20**		**F21**		**F22**	
**Algo.**	**Avg**	**Std**	**Avg**	**Std**	**Avg**	**Std**
CLFGO	$2.1684\times 10^{3}$	$9.9530\times 10^{1}$	$2.3665\times 10^{3}$	$1.4913\times 10^{1}$	$2.8348\times 10^{3}$	$1.0140\times 10^{3}$
FGO	$2.5064\times 10^{3}$	$1.2560\times 10^{2}$	$2.4777\times 10^{3}$	$3.9610\times 10^{1}$	$2.3010\times 10^{3}$	$1.6392\times 10^{0}$
BA	$2.9968\times 10^{3}$	$1.8068\times 10^{2}$	$2.6322\times 10^{3}$	$7.6396\times 10^{1}$	$6.5599\times 10^{3}$	$1.8544\times 10^{3}$
MGO	$2.2551\times 10^{3}$	$7.7499\times 10^{1}$	$2.3719\times 10^{3}$	$1.2168\times 10^{1}$	$2.8287\times 10^{3}$	$1.3438\times 10^{3}$
PO	$2.4920\times 10^{3}$	$1.7183\times 10^{2}$	$2.5003\times 10^{3}$	$4.1516\times 10^{1}$	$3.0979\times 10^{3}$	$1.6947\times 10^{3}$
DE	$2.1425\times 10^{3}$	$7.2484\times 10^{1}$	$2.4094\times 10^{3}$	$9.0707\times 10^{0}$	$4.2000\times 10^{3}$	$2.0147\times 10^{3}$
HGS	$2.5202\times 10^{3}$	$2.1445\times 10^{2}$	$2.4286\times 10^{3}$	$3.1083\times 10^{1}$	$4.9980\times 10^{3}$	$1.2853\times 10^{3}$
GWO	$2.4200\times 10^{3}$	$1.5127\times 10^{2}$	$2.3859\times 10^{3}$	$2.3691\times 10^{1}$	$4.4522\times 10^{3}$	$1.3513\times 10^{3}$
SMA	$2.4322\times 10^{3}$	$1.3670\times 10^{2}$	$2.4751\times 10^{3}$	$2.5228\times 10^{1}$	$3.4253\times 10^{3}$	$1.6710\times 10^{3}$
SCA	$2.6059\times 10^{3}$	$1.0097\times 10^{2}$	$2.5559\times 10^{3}$	$2.2058\times 10^{1}$	$8.3144\times 10^{3}$	$2.2804\times 10^{3}$
	**F23**		**F24**		**F25**	
**Algo.**	**Avg**	**Std**	**Avg**	**Std**	**Avg**	**Std**
CLFGO	$2.7190\times 10^{3}$	$1.5028\times 10^{1}$	$2.9410\times 10^{3}$	$3.2951\times 10^{1}$	$2.8861\times 10^{3}$	$2.5847\times 10^{0}$
FGO	$3.0667\times 10^{3}$	$9.5498\times 10^{1}$	$3.2420\times 10^{3}$	$1.2636\times 10^{2}$	$2.9282\times 10^{3}$	$2.1691\times 10^{1}$
BA	$3.3586\times 10^{3}$	$1.5560\times 10^{2}$	$3.3492\times 10^{3}$	$1.0859\times 10^{2}$	$2.9017\times 10^{3}$	$1.8713\times 10^{1}$
MGO	$2.7204\times 10^{3}$	$1.4945\times 10^{1}$	$2.8981\times 10^{3}$	$1.2466\times 10^{1}$	$2.8874\times 10^{3}$	$9.1349\times 10^{-1}$
PO	$2.9415\times 10^{3}$	$7.1979\times 10^{1}$	$3.1225\times 10^{3}$	$6.4519\times 10^{1}$	$2.9347\times 10^{3}$	$2.4053\times 10^{1}$
DE	$2.7572\times 10^{3}$	$8.5421\times 10^{0}$	$2.9542\times 10^{3}$	$1.3829\times 10^{1}$	$2.8873\times 10^{3}$	$3.1655\times 10^{-1}$
HGS	$2.7778\times 10^{3}$	$2.7694\times 10^{1}$	$3.0268\times 10^{3}$	$6.2773\times 10^{1}$	$2.8910\times 10^{3}$	$1.2417\times 10^{1}$
GWO	$2.7535\times 10^{3}$	$3.2826\times 10^{1}$	$2.9216\times 10^{3}$	$4.7920\times 10^{1}$	$2.9830\times 10^{3}$	$5.8611\times 10^{1}$
SMA	$2.8523\times 10^{3}$	$3.0503\times 10^{1}$	$3.0201\times 10^{3}$	$3.1537\times 10^{1}$	$2.9933\times 10^{3}$	$3.8583\times 10^{1}$
SCA	$2.9984\times 10^{3}$	$2.7915\times 10^{1}$	$3.1611\times 10^{3}$	$3.1751\times 10^{1}$	$3.1737\times 10^{3}$	$5.3460\times 10^{1}$
	**F26**		**F27**		**F28**	
**Algo.**	**Avg**	**Std**	**Avg**	**Std**	**Avg**	**Std**
CLFGO	$3.9654\times 10^{3}$	$6.7275\times 10^{2}$	$3.2114\times 10^{3}$	$9.2192\times 10^{0}$	$3.1472\times 10^{3}$	$6.0208\times 10^{1}$
FGO	$5.1451\times 10^{3}$	$2.2654\times 10^{3}$	$3.5693\times 10^{3}$	$1.3619\times 10^{2}$	$3.2151\times 10^{3}$	$2.9918\times 10^{1}$
BA	$8.2947\times 10^{3}$	$2.5934\times 10^{3}$	$3.4247\times 10^{3}$	$1.5669\times 10^{2}$	$3.1332\times 10^{3}$	$5.7901\times 10^{1}$
MGO	$3.9685\times 10^{3}$	$4.6393\times 10^{2}$	$3.2126\times 10^{3}$	$4.8143\times 10^{0}$	$3.2339\times 10^{3}$	$1.4265\times 10^{1}$
PO	$6.3802\times 10^{3}$	$1.9104\times 10^{3}$	$3.3019\times 10^{3}$	$3.9899\times 10^{1}$	$3.3119\times 10^{3}$	$3.8727\times 10^{1}$
DE	$4.6657\times 10^{3}$	$1.0047\times 10^{2}$	$3.2060\times 10^{3}$	$3.3728\times 10^{0}$	$3.1900\times 10^{3}$	$4.6866\times 10^{1}$
HGS	$4.9201\times 10^{3}$	$2.5296\times 10^{2}$	$3.2258\times 10^{3}$	$1.3544\times 10^{1}$	$3.2167\times 10^{3}$	$3.8900\times 10^{1}$
GWO	$4.5536\times 10^{3}$	$4.0377\times 10^{2}$	$3.2533\times 10^{3}$	$2.5744\times 10^{1}$	$3.4435\times 10^{3}$	$1.5892\times 10^{2}$
SMA	$5.1881\times 10^{3}$	$6.9145\times 10^{2}$	$3.2530\times 10^{3}$	$2.4309\times 10^{1}$	$3.4201\times 10^{3}$	$6.2090\times 10^{1}$
SCA	$7.0468\times 10^{3}$	$2.4922\times 10^{2}$	$3.4033\times 10^{3}$	$3.9376\times 10^{1}$	$3.8630\times 10^{3}$	$1.3177\times 10^{2}$
	**F29**		**F30**			
**Algo.**	**Avg**	**Std**	**Avg**	**Std**		
CLFGO	$3.4181\times 10^{3}$	$1.0825\times 10^{2}$	$5.1623\times 10^{3}$	$1.8983\times 10^{2}$		
FGO	$4.4539\times 10^{3}$	$3.6476\times 10^{2}$	$1.6062\times 10^{5}$	$1.5679\times 10^{5}$		
BA	$5.0101\times 10^{3}$	$5.9578\times 10^{2}$	$1.2658\times 10^{6}$	$7.8956\times 10^{5}$		
MGO	$3.6333\times 10^{3}$	$9.4229\times 10^{1}$	$6.1726\times 10^{4}$	$3.0469\times 10^{4}$		
PO	$4.4419\times 10^{3}$	$3.5618\times 10^{2}$	$6.9220\times 10^{6}$	$5.1619\times 10^{6}$		
DE	$3.5130\times 10^{3}$	$7.5662\times 10^{1}$	$1.1489\times 10^{4}$	$2.7288\times 10^{3}$		
HGS	$3.7876\times 10^{3}$	$1.8836\times 10^{2}$	$5.8306\times 10^{4}$	$7.6510\times 10^{4}$		
GWO	$3.7556\times 10^{3}$	$1.5097\times 10^{2}$	$7.0190\times 10^{6}$	$8.4021\times 10^{6}$		
SMA	$4.0269\times 10^{3}$	$2.8186\times 10^{2}$	$4.4078\times 10^{6}$	$3.6517\times 10^{6}$		
SCA	$4.6254\times 10^{3}$	$2.0776\times 10^{2}$	$6.7893\times 10^{7}$	$2.9522\times 10^{7}$		
**Overall Rank**						
**Algo.**	**RANK**	**+/=/−**	**AVG**			
CLFGO	1	∼	1.5517			
FGO	5	27/1/1	5.3103			
BA	9	27/2/0	7.4483			
MGO	3	17/11/1	3.3448			
PO	8	28/1/0	7.1034			
DE	2	22/4/3	3.3103			
HGS	4	29/0/0	4.6552			
GWO	6	28/0/1	6.0345			
SMA	7	29/0/0	7.0345			
SCA	10	29/0/0	9.2069			

**Table 8:** The *p*-values of the CLFGO versus other algorithms on CEC2017.

Fun	BA	MGO	PO	FGO	DE	HGS	GWO	SMA	SCA
F1	$1.73\times 10^{-6}$	$1.73\times 10^{-6}$	$1.73\times 10^{-6}$	$1.73\times 10^{-6}$	$1.73\times 10^{-6}$	$1.73\times 10^{-6}$	$1.73\times 10^{-6}$	$1.73\times 10^{-6}$	$1.73\times 10^{-6}$
F3	$2.71\times 10^{-1}$	$1.73\times 10^{-6}$	$1.73\times 10^{-6}$	$9.32\times 10^{-6}$	$1.73\times 10^{-6}$	$1.73\times 10^{-6}$	$1.73\times 10^{-6}$	$1.73\times 10^{-6}$	$1.73\times 10^{-6}$
F4	$2.16\times 10^{-5}$	$3.52\times 10^{-6}$	$1.73\times 10^{-6}$	$1.92\times 10^{-6}$	$1.73\times 10^{-6}$	$2.60\times 10^{-6}$	$1.73\times 10^{-6}$	$1.73\times 10^{-6}$	$1.73\times 10^{-6}$
F5	$1.73\times 10^{-6}$	$6.88\times 10^{-1}$	$1.73\times 10^{-6}$	$1.73\times 10^{-6}$	$1.92\times 10^{-6}$	$4.73\times 10^{-6}$	$7.69\times 10^{-6}$	$1.73\times 10^{-6}$	$1.73\times 10^{-6}$
F6	$1.73\times 10^{-6}$	$9.59\times 10^{-1}$	$1.73\times 10^{-6}$	$1.73\times 10^{-6}$	$1.73\times 10^{-6}$	$1.73\times 10^{-6}$	$1.73\times 10^{-6}$	$1.73\times 10^{-6}$	$1.73\times 10^{-6}$
F7	$1.73\times 10^{-6}$	$1.25\times 10^{-4}$	$1.73\times 10^{-6}$	$1.73\times 10^{-6}$	$1.73\times 10^{-6}$	$1.73\times 10^{-6}$	$1.73\times 10^{-6}$	$1.73\times 10^{-6}$	$1.73\times 10^{-6}$
F8	$1.73\times 10^{-6}$	$2.71\times 10^{-1}$	$1.73\times 10^{-6}$	$1.73\times 10^{-6}$	$1.73\times 10^{-6}$	$1.92\times 10^{-6}$	$4.90\times 10^{-4}$	$1.73\times 10^{-6}$	$1.73\times 10^{-6}$
F9	$1.73\times 10^{-6}$	$6.29\times 10^{-1}$	$1.73\times 10^{-6}$	$1.73\times 10^{-6}$	$1.73\times 10^{-6}$	$1.73\times 10^{-6}$	$1.92\times 10^{-6}$	$1.73\times 10^{-6}$	$1.73\times 10^{-6}$
F10	$1.73\times 10^{-6}$	$1.73\times 10^{-6}$	$1.73\times 10^{-6}$	$1.73\times 10^{-6}$	$1.73\times 10^{-6}$	$1.36\times 10^{-5}$	$1.92\times 10^{-6}$	$1.73\times 10^{-6}$	$1.73\times 10^{-6}$
F11	$1.73\times 10^{-6}$	$1.80\times 10^{-5}$	$1.73\times 10^{-6}$	$1.92\times 10^{-6}$	$4.39\times 10^{-3}$	$1.36\times 10^{-5}$	$1.73\times 10^{-6}$	$1.73\times 10^{-6}$	$1.73\times 10^{-6}$
F12	$1.73\times 10^{-6}$	$1.73\times 10^{-6}$	$1.73\times 10^{-6}$	$1.73\times 10^{-6}$	$1.73\times 10^{-6}$	$1.73\times 10^{-6}$	$1.73\times 10^{-6}$	$1.73\times 10^{-6}$	$1.73\times 10^{-6}$
F13	$1.73\times 10^{-6}$	$1.73\times 10^{-6}$	$1.73\times 10^{-6}$	$1.73\times 10^{-6}$	$1.73\times 10^{-6}$	$1.73\times 10^{-6}$	$1.73\times 10^{-6}$	$1.73\times 10^{-6}$	$1.73\times 10^{-6}$
F14	$1.73\times 10^{-6}$	$1.73\times 10^{-6}$	$1.73\times 10^{-6}$	$1.73\times 10^{-6}$	$1.73\times 10^{-6}$	$1.73\times 10^{-6}$	$1.73\times 10^{-6}$	$1.73\times 10^{-6}$	$1.73\times 10^{-6}$
F15	$1.73\times 10^{-6}$	$1.73\times 10^{-6}$	$1.73\times 10^{-6}$	$1.73\times 10^{-6}$	$1.73\times 10^{-6}$	$1.73\times 10^{-6}$	$1.73\times 10^{-6}$	$1.73\times 10^{-6}$	$1.73\times 10^{-6}$
F16	$1.73\times 10^{-6}$	$4.41\times 10^{-1}$	$1.73\times 10^{-6}$	$1.73\times 10^{-6}$	$6.56\times 10^{-2}$	$2.88\times 10^{-6}$	$5.67\times 10^{-3}$	$1.73\times 10^{-6}$	$1.73\times 10^{-6}$
F17	$1.73\times 10^{-6}$	$1.16\times 10^{-1}$	$1.92\times 10^{-6}$	$1.92\times 10^{-6}$	$5.86\times 10^{-1}$	$4.29\times 10^{-6}$	$1.11\times 10^{-3}$	$3.18\times 10^{-6}$	$1.73\times 10^{-6}$
F18	$1.73\times 10^{-6}$	$1.73\times 10^{-6}$	$1.73\times 10^{-6}$	$1.73\times 10^{-6}$	$1.73\times 10^{-6}$	$1.73\times 10^{-6}$	$1.73\times 10^{-6}$	$1.73\times 10^{-6}$	$1.73\times 10^{-6}$
F19	$1.73\times 10^{-6}$	$1.73\times 10^{-6}$	$1.73\times 10^{-6}$	$1.73\times 10^{-6}$	$1.73\times 10^{-6}$	$1.73\times 10^{-6}$	$1.73\times 10^{-6}$	$1.73\times 10^{-6}$	$1.73\times 10^{-6}$
F20	$1.73\times 10^{-6}$	$3.61\times 10^{-3}$	$2.60\times 10^{-6}$	$1.73\times 10^{-6}$	$3.39\times 10^{-1}$	$3.52\times 10^{-6}$	$9.32\times 10^{-6}$	$2.35\times 10^{-6}$	$1.73\times 10^{-6}$
F21	$1.73\times 10^{-6}$	$3.93\times 10^{-1}$	$1.73\times 10^{-6}$	$1.73\times 10^{-6}$	$1.92\times 10^{-6}$	$1.73\times 10^{-6}$	$5.71\times 10^{-4}$	$1.73\times 10^{-6}$	$1.73\times 10^{-6}$
F22	$3.88\times 10^{-6}$	$1.41\times 10^{-1}$	$1.06\times 10^{-1}$	$6.14\times 10^{-1}$	$8.73\times 10^{-3}$	$1.24\times 10^{-5}$	$3.59\times 10^{-4}$	$4.49\times 10^{-2}$	$2.60\times 10^{-6}$
F23	$1.73\times 10^{-6}$	$6.58\times 10^{-1}$	$1.73\times 10^{-6}$	$1.73\times 10^{-6}$	$1.73\times 10^{-6}$	$2.60\times 10^{-6}$	$3.72\times 10^{-5}$	$1.73\times 10^{-6}$	$1.73\times 10^{-6}$
F24	$1.73\times 10^{-6}$	$3.88\times 10^{-6}$	$1.73\times 10^{-6}$	$1.73\times 10^{-6}$	$5.19\times 10^{-2}$	$2.88\times 10^{-6}$	$2.70\times 10^{-2}$	$6.98\times 10^{-6}$	$1.73\times 10^{-6}$
F25	$1.13\times 10^{-5}$	$1.38\times 10^{-3}$	$1.73\times 10^{-6}$	$1.73\times 10^{-6}$	$2.83\times 10^{-4}$	$3.87\times 10^{-2}$	$1.73\times 10^{-6}$	$1.73\times 10^{-6}$	$1.73\times 10^{-6}$
F26	$6.98\times 10^{-6}$	$8.13\times 10^{-1}$	$1.80\times 10^{-5}$	$8.73\times 10^{-3}$	$2.35\times 10^{-6}$	$1.92\times 10^{-6}$	$6.89\times 10^{-5}$	$5.22\times 10^{-6}$	$1.73\times 10^{-6}$
F27	$1.92\times 10^{-6}$	$7.50\times 10^{-1}$	$1.73\times 10^{-6}$	$1.73\times 10^{-6}$	$1.48\times 10^{-3}$	$2.41\times 10^{-4}$	$1.73\times 10^{-6}$	$1.73\times 10^{-6}$	$1.73\times 10^{-6}$
F28	$8.29\times 10^{-1}$	$4.73\times 10^{-6}$	$1.73\times 10^{-6}$	$2.41\times 10^{-4}$	$9.77\times 10^{-3}$	$1.25\times 10^{-4}$	$1.73\times 10^{-6}$	$1.73\times 10^{-6}$	$1.73\times 10^{-6}$
F29	$1.73\times 10^{-6}$	$6.34\times 10^{-6}$	$1.73\times 10^{-6}$	$1.73\times 10^{-6}$	$5.31\times 10^{-5}$	$2.88\times 10^{-6}$	$1.92\times 10^{-6}$	$1.73\times 10^{-6}$	$1.73\times 10^{-6}$
F30	$1.73\times 10^{-6}$	$1.73\times 10^{-6}$	$1.73\times 10^{-6}$	$1.73\times 10^{-6}$	$1.73\times 10^{-6}$	$1.73\times 10^{-6}$	$1.73\times 10^{-6}$	$1.73\times 10^{-6}$	$1.73\times 10^{-6}$

**Table 9:** Experimental Results of All Algorithms on the Production Optimization Problem.

Algorithm	Mean (USD)	Std	Best (USD)	Worst (USD)
CLFGO	$9.9742\times 10^{8}$	$1.353\times 10^{7}$	$1.046\times 10^{9}$	$9.785\times 10^{8}$
DE	$9.536\times 10^{8}$	$1.845\times 10^{7}$	$9.721\times 10^{8}$	$9.348\times 10^{8}$
MGO	$9.487\times 10^{8}$	$1.954\times 10^{7}$	$9.680\times 10^{8}$	$9.292\times 10^{8}$
HGS	$9.321\times 10^{8}$	$2.164\times 10^{7}$	$9.532\times 10^{8}$	$9.075\times 10^{8}$
FGO	$9.145\times 10^{8}$	$2.512\times 10^{7}$	$9.427\times 10^{8}$	$8.885\times 10^{8}$
PO	$9.043\times 10^{8}$	$2.737\times 10^{7}$	$9.349\times 10^{8}$	$8.798\times 10^{8}$
BA	$8.921\times 10^{8}$	$2.981\times 10^{7}$	$9.262\times 10^{8}$	$8.656\times 10^{8}$
GWO	$8.762\times 10^{8}$	$3.128\times 10^{7}$	$9.150\times 10^{8}$	$8.437\times 10^{8}$
SMA	$8.524\times 10^{8}$	$3.487\times 10^{7}$	$8.906\times 10^{8}$	$8.192\times 10^{8}$
SCA	$8.241\times 10^{8}$	$4.156\times 10^{7}$	$8.734\times 10^{8}$	$7.821\times 10^{8}$

## Data Availability

The numerical and experimental data used to support the findings of this study are included within the article.

## Abbreviations

The following abbreviations are used in this manuscript:

CLFGO	Comprehensive Learning Fungal Growth Optimizer
FGO	Fungal Growth Optimizer
CL	Comprehensive Learning
NPV	Net Present Value
BHP	Bottom-Hole Pressure
RPO	Reservoir Production Optimization
CEC	Congress on Evolutionary Computation
DE	Differential Evolution
HGS	Hunger Games Search
PSO	Particle Swarm Optimization
SMA	Slime Mould Algorithm
PO	Parrot Optimizer
BA	Bat Algorithm
MGO	Moss Growth Optimization
GWO	Grey Wolf Optimizer
SCA	Sine Cosine Algorithm
NFL	No Free Lunch
